# Essays on the Most Important Diseases of Women

**Published:** 1839-04

**Authors:** 


### Art. IX.

*Essays on the most important Diseases of Women.* By Robert Ferguson, m.d., Fellow of the Royal College of Physicians, London; Professor of Obstetric Medicine and Diseases of Women and Children, King's College, London; Senior Physician to the General Lying-in Hospital. Part I. Puerperal Fever.—*London*, 1839. 8vo. pp. 299.

We believe that the impression left on the minds of reflecting practitioners, by a consideration of the various kinds of treatment reported as successful in cases of puerperal fever, has long been that the affections described by different writers, and reported to be successfully treated by very opposite (sometimes by diametrically opposite) methods, could not be strictly identical; and that, if all the cases so denominated were really cases of puerperal fever, they differed in being accompanied or not by inflammation in various degrees, and perhaps of various organs; whilst, as in other fevers, different epidemics were characterized by different types, ranging from the acutely inflammatory to the typhoid. There appeared, also, every reason to believe that, in some of the cases recorded under the general head of puerperal fever, the affection was not only not inflammatory, but not febrile; being essentially a state of nervous commotion, combined with spasm or neuralgia, incidental to the puerperal condition. In a notice of Mr. Moore's work, in our Second Volume, we ventured to express our own belief that the affections which we ourselves considered as especially entitled to the name of puerperal fever belonged to that class of diseases the fundamental character of which is a morbid condition of the blood, produced by the introduction of some deleterious agent into the circulation. Some of the conclusions to which we have alluded derived confirmation, or certainly support, from Dr. Gooch's clear and candid statements; although the views of some previous and some subsequent authorities have been directed to such inflammatory complications of puerperal fever as render the appellation of peritoneal fevers, adopted by Dr. Gooch, of at least very doubtful correctness. The subject, upon the whole, was left, by previous writers, exactly in such a state as made it desirable that some practitioner of learning and of great experience, and gifted with a clear understanding and calm judgment, would altogether revise it, and produce order out of what was little better than confusion. We almost ventured to prophesy, in another article in the volume of this Journal already referred to, (notice of Dr. Alexander's Commentaries,) that the great establishments of London or Dublin would furnish such an one. Precisely such an author has presented himself in the person of the distinguished professor in King's College; and, although we shall take the liberty of reviewing his opinions with our customary freedom, we enter upon the task with a strong belief that, in the general tenor of his doctrines, he will be found at once comprehensive and exact. We observe that he is deeply impressed with a sense of the intricacy in which both the theory and practice of this disease are involved; a disease so serious as to occasion "seven-eighths of the total mortality in childbirth:" in support of this and other statements, the author is fortunately able to appeal to the valuable materials furnished by cases occurring in the General Lying-in Hospital in the last twelve years, 204 in number; four fifths of which he either treated himself or witnessed the treatment of by others.

Dr. Ferguson commences his work with the following passages, which briefly and clearly explain the view he takes of the various forms of the disease.

"I have observed four different forms of puerperal fever, each possessing very distinct general characters, and requiring for its cure a distinct treatment. The first may be termed the 'peritoneal form,' as the intensity of the malady seems to be expended on the peritoneum, and lingers longest in it. In the second the general course of the disease is that of common fever, marked by much disorder of the abdominal viscera. Very often the symptoms are those of remittent fevers, and occasionally of intermittents. In the third form the brain and nervous system seem especially affected; while the fourth, which may be termed the 'complicated,' embraces the lesions and symptoms of the other three, with this remarkable feature in addition, a tendency to fluid deposition in any or in every tissue. The fluids effused are blood, serum, pus, and lymph. This last form has always excited the most attention; for it is the most frequent and the most fatal, and, under circumstances of crowded hospitals, and misery, and poverty, intractable to remedial measures.

"These four forms—the peritoneal, the gastro-enteric, the nervous, and the complicated,—spring, as I shall endeavour to show, from one source and cause. There is no trenchant limit which bounds them in nature; and, in every epidemic which I have witnessed, the characteristics peculiar to any one are readily assumed by another. In the peritoneal form, the peritonitis ceases, and the patient succumbs under fever and diarrhœa, or under a complication of local effusions and phlegmasiæ. In the gastro-enteric, sudden peritonitis will supervene, and carry off the patient in a few hours. Such also may occur in the third or nervous form, or it may (though it rarely does) terminate by a lengthened fever, or by deposit in the great cavities, or in the joints, the muscles, or the eyeballs. It is the last form alone which is invariable, save in intensity. It may be looked on as a summary of the others, while these may be considered as fragments of it.

"Although these various forms may and do run into each other, their tendency is to remain distinct; and it is a remarkable fact that the one or the other of these will give the character to a whole epidemic; the rest either totally disappearing or appearing only rarely. It is this which has caused so much confusion and dispute in the history of opinions on puerperal fever. Each author has assumed the form he witnessed as the only one, to the exclusion of all the rest. Hence, too, the discrepancy in the treatment of this malady. Gordon, and Armstrong, and Hey saw, for the most part, the peritoneal form. William Hunter, Lowder, and the elder Hey, observed chiefly the complicated form, where the whole constitution was sinking under numerous attacks, simultaneously made, on the most distant organs in every grade of intensity. Richter and Butter witnessed epidemics in which the gastro-enteric form predominated, and in which the disordered secretions were remedied by ipecacuanha or alkalies, followed by stimulants." (p. 1.)

Further confusion has arisen, Dr. Ferguson observes, from the various intensity in which any of these forms of the puerperal fever may exist; in consequence of which, the treatment proper in one epidemic is found to be inefficacious in the next. He carefully compares his division with the divisions described, or the peculiar forms witnessed, by Douglas, Tonellé, Ed. Martens, Clark, Vigarous, Gardien, Gooch, Dugés, Boivin, Blundell, Busch, Ritgen, Robert Lee, Stoll, Doulcet, Tricher, Gasc, Murat, Gordon, Hey, Armstrong, Alphonse le Roy, John Clarke, William Hunter, the elder Hey, and Campbell; in order to show that, where any of these observers have given more than four forms, the forms seem reducible under one of the four which he has adopted, and that each of the single and peculiar forms witnessed by the rest, as by

Gordon and Armstrong for instance, were in reality one of the four which he describes.

Of these four forms of puerperal fever a minute and careful description is given, illustrated by cases; and the distinctions are of so much importance that we shall endeavour to condense the descriptions under their respective headings.

*First form of puerperal fever, characterized by abdominal pain.* Of this there are two kinds; one durable and dangerous, the other transient; but the distinction between the two in the commencement of an epidemic is very difficult. In both there is intense pain between the pubes and a line drawn from the crest of one ilium to that of the other; in both the attacks are ushered in by rigor; both occur about the same period, from the first to the fifth day after parturition; nor are they distinguishable by the degree of the fever or by the state of the pulse. This statement is calculated to produce great anxiety in the practitioner's mind; and it is difficult to see how mistake and danger can be avoided until the only test, that of the remedies, has been tried; when it is found that the transitory form is relieved by agents which lull the pain, and that the severer form demands the remedies of pure inflammation. The means of immediate diagnosis rarely exist. The transient form may pass on to the severer. Neither Mr. Griffin's supposed pathognomic sign, that when there is inflammation of the peritoneum the patient cannot stretch the limbs, nor the peculiarly small and compressed pulse, to which Mr. Hingeston attaches weight, are always wanting in the transient and uninflammatory form. If the attack is sudden, and followed by intermission or marked remission; if the pain be constant, and yet the nature of the epidemic shows it to have been easily removed in other cases; its transient character may however be presumed, and, as will be shown by and by, safely acted upon. Two cases illustrating the severe and deceptious symptoms of the transitory form, and their complete relief by Dover's powder and aperients, are selected from those published by Mr. Hingeston, and observed by him in the General Lying-in Hospital. A case is also quoted as published by Mr. Griffin, in the Medical Gazette for October 15, 1836, in which alarming symptoms were combated by aperients and opium. It is highly probable, as Dr. Ferguson supposes, that these were the cases in which Doulcet, Richter, and Boer produced cures by medicines which might be deemed insignificant when compared with the apparent severity of the disease with which they had to contend. The preparation employed with such alleged advantage by Boer, as mentioned in Dr. Gooch's work, and of which the secret was not divulged, is concluded by Dr. Ferguson, from a perusal of the last edition of Boer's work, to have been the "true James's powder," and not the Kermes mineral, as Dr. Gooch suspected.

Alluding to Mr. Hingeston's opinion that this first form is nervous, in the sense in which a nervous impression precedes every inflammation, the peculiarity in this instance being its permanency in this early stage; and also to the view entertained of it by Mr. Griffin, as being a nervous pain dependent on spinal irritation; and that of M. Tonellé, that it is connected with the circulation of pus in the veins; Dr. Ferguson observes,

"I believe these opinions are not really different, except in each being only a part of the truth. M. Tonellé, I think, and shall prove hereafter, would have given the real cause had he generalized his proposition into a vitiated state of the blood, instead of limiting such vitiation to one cause, namely, the circulation of pus. In many instances we have the genuine marks of vitiated fluids, without being able to detect a particle of pus in them. Le Gallois mixed pus with blood, while it flowed from the arm, but could not subsequently detect in the compound any of the matter: hence pus may exist in the circulation without our being able to discover it. But I shall hereafter show that other products of inflammation will vitiate the blood. I would, however, strongly impress the experiment of Le Gallois on the reader. He will hereafter find many cases exhibiting the genuine character of phlebitis, in which no lesion of the vein was discoverable, and which this experiment explains, and, as many other sources of vitiation exist, to select one is exclusive, and may be erroneous.

"Admitting, with Tonellé, that a vitiated blood is circulating in the system, the two opinions of Mr. Hingeston and Mr. Griffin are readily reconcileable; the latter tracing the peritoneal pain to an irritating cause, acting on the spine first, and thence on the peritoneum; the former merely delineating the disease as a nervous affection of the peritoneum, the commencement of true inflammation, without tracing the affection to the nervous centres themselves. In all, we must conclude that the malady is a genuine puerperal fever, the peculiarity of which consists in its being limited to its very first stage; consequently, in its being within the reach of such simple remedies as merely allay pain and subdue congestion, by promoting the secretions of the intestinal canal and the skin. My own experience, and the inferences derivable from the perusal of Doulcet, Richter, and Boer, prove it is not a matter of indifference how such a malady should be treated. I believe that a severe bleeding will give to that which was transitory a permanent character, and what might have been easily removed by appropriate remedies becomes now a formidable disease." (p. 13.)

In many instances of this first form of puerperal fever, a hot linseed-meal poultice is often alone sufficient to give relief; an opiate may then be given, with or without an aperient; but, if no relief follows by this method, it is not deemed safe to wait more than four, or at the utmost six hours, without changing the treatment. The severer affection is a genuine peritonitis, and requires the energetic treatment adapted to that affection. There are four distinct stages; a preparatory stage of shivering, heat, perspiration, and anxiety of mind; then pain, fever, a hard, frequent, and compressed pulse, "the supine posture, and restrained breathing;" then the stage of effusion, with an apparent amelioration of the symptoms; to which succeeds the stage of collapse. It is found, generally speaking, that inflammation of the peritoneum only is of rare occurrence.

*Second form of puerperal fever: fever with gastro-enteric irritation.* In this form there is either no peritoneal affection, or it is slight and soon lost amidst other constitutional symptoms. The disease has the character of mild typhus, with intestinal irritation; and rarely lasts less than seven or more than twenty days. It rarely or never becomes fatal without being complicated with acute inflammation of some important organ, as the peritoneum or the thoracic viscera; or some deposition in the joints or limbs, followed by colliquative diarrhœa. The uterus is found either much congested and large, or pus is formed in its veins and lymphatics, or there is superficial softening of its inner mucous surface.

"When there is extensive softening of this viscus, all the above characteristics of this form of puerperal disease are merged in the broad and very obvious signs of an overwhelming, and from the very first a fatal typhus. There is no peritoneal pain, but merely very deep-seated and obtuse sensibility to firm pressure. The mind and body are equally prostrate and enfeebled. The skin is of a deep brown colour; the pulse small, weak, and very rapid; the abdomen soon becomes tympanitic, and the whole intestinal canal seems filled with dark fluid, which is vomited without effort in large gushes, or passed in unrestrainable diarrhœa." (p. 23.)

*Third or nervous form.* In this variety, the rarest of the four in its pure form, although often seen for a time to change the character of the other forms, there is painful and sudden abdominal tenderness, which quickly subsides; the pulse is rapid, there is great restlessness, mental agitation, and "shifting functional disturbance of various organs; sighings, tremors, and cramps; sudden and deathlike sinking, and as sudden reappearance of strength." Terror is a frequent accompaniment of this form; furious delirium often ushers it in; and fatal coma or sudden syncope supervene.

*Fourth or complicated form of puerperal fever.* "This form," says Dr. Ferguson, (to whose graphic expressions we are continually inclined to give the preference,)

"This form is characterized by an appalling complication of symptoms, arising from the simultaneous or the rapidly successive attacks on distant organs and differing tissues. It begins on the first to the third day, with shivering, which very often, though not always, is followed by abdominal pain. The debility soon amounts to complete prostration. The mind is calm, and often wonderfully unconscious of danger. There is the rapid pulse and the dusky skin of the second form, and the crimson patches on the cheeks, which contrast so peculiarly with the dull glassy eye, encircled in lead-coloured lids. Sometimes the abdominal pain lasts during the whole of the brief period of the malady; sometimes it subsides in a few hours. Shortly after the abdominal attack, other organs begin to exhibit signs of disorder." (p. 26.)

We have no doubt that the practitioner may very conveniently keep this fourfold division in view, although we can imagine that the third variety is not always distinguishable from one of the forms of the first; nor even the fourth from the second. But the forms are evidently based upon important differences in the character of the malady in different cases, and their recognition appears calculated to suggest practical considerations which are important. When this last form becomes secondarily complicated, the intestinal canal usually becomes the seat of disorder; there is diarrhœa, going on to dysentery; or nausea, going on to coffee-coloured vomiting. The function of respiration is almost always disturbed; "there is sighing, loud, incessant, and automatic, independent on mental and bodily anguish;" and sometimes the lungs become inflamed, or even gangrenous. The pleura, the heart, the pericardium, the œsophagus, may become the seat of disease; inflammation of the pleura or pericardium, and effusion; softening of the heart, gangrene of the œsophagus, have been observed; perforation of the stomach or intestines may take place; and the cellular and muscular tissue of the limbs may become affected with what at first appears to be only acute rheumatism, and effusions of pus, serum, or blood, or of all, may follow, without relief, except in the milder cases. These last results are most to be expected on the back of the forearm, and on the calves of the legs near a joint; and they occur so suddenly as to have been supposed independent on an inflammatory process. The joints are often similarly affected. Dr. Ferguson has also noticed two other states of the limbs connected with puerperal fever; one resembling erysipelas, and the other, always in the leg, phlegmasia dolens. The connexion between erysipelas and puerperal fever is, he says, illustrated by the fact that the two maladies are generally coexistent in the hospital, and "when the mothers die of puerperal fevers, their infants perish of erysipelas:" and, with respect to phlegmasia dolens, he subscribes to the opinion of Le Gallois and Professor Busch, of Berlin, that it is only puerperal fever modified by its locality. In most cases of this last disorder the vein is thickened, but not in all; and Dr. F. adopts the opinion that pus in the lymphatics will produce the same effects as when circulating in a vein. Much of the pain, he remarks, arises, where the swelling is inconsiderable, from inflammation of the nervous trunks, "which are clustered over with minute vessels, and are redder in colour." He has not seen this appearance in the nerves running over the peritoneum, so repeatedly mentioned by Campbell.

To add to the formidable character of this complicated form of puerperal fever, it is sometimes accompanied with what may be called the gangrenous diathesis. Dr. Ferguson has known the calf of the leg become black and gangrenous in four hours; but the parts most usually affected are the vagina, vulva, and sacral region. The eye is sometimes the seat of this affection; and there is even hazard of blindness in those in whom the suffusion is only slight, but accompanied by burning pain in the ball. Purulent effusion into the eye and its total destruction are not uncommon.

"When this fourth form of puerperal fever is prevalent," says Dr. Ferguson, "and the characteristic of the epidemic, one in three dies in the London hospitals, I believe, under any treatment; and, if the complications be many, this mortality will be still increased, and the most judicious and the most humane act is to shut up these receptacles forthwith. Such is the experience of William Hunter,—of those who have witnessed its plague-like ravages in the continental hospitals,—and such is mine. Happily, however, this form of puerperal fever exists with such fatality in hospitals alone. I believe that the single chamber of the pauper is more wholesome than the spacious ward of the hospital patient; while in private practice, among the well-housed and well nourished, the milder forms are the commonest. In this form we find great variations of intensity of attack, as well as in the grouping of organs attacked. Different epidemics present singular diversities in these two particulars. In one year there are no muscular abscesses, in another no uterine softening; and yet the generic characters and the main features in both are the same. Tenon and the elder French authors give curious examples of the prevalence of certain symptoms in one year, and their omission in another. (*Baudeloque.*) As to the organs attacked, taken isolatedly, my experience proves to me, 1. That each organ may undergo every grade of disease, from simple irritation, which will not proceed further, to total destruction and softening of its tissues. 2. That when many organs are attacked at once, or consecutively, they are not wrought up to one uniform pitch of malady; that while one is simply irritated, another is gangrenous. Thus, I have seen coexistent inflammation of the peritoneum, with painless and sudden perforation of the œsophagus. What are our indications for such a disease, and what our hopes of cure?" (p. 31.)

These ample and clear descriptions convey, perhaps, a juster idea of the disease called puerperal fever, in the various shapes it assumes in different cases and different epidemics, than it was previously possible to gather from conflicting testimonies; and to prepare the reader to admit the general theory of the disease professed by Dr. Ferguson. Although we trust every student will omit no part of the instructive work before us, we shall ourselves pass over the chapter on the Morbid Anatomy with little remark of our own; believing that this part of the subject, the study of which is doubtless extremely important, has been cultivated with a diligence more than proportionate to the reflection bestowed either on the nature or the treatment of this formidable and changeful malady.

The morbid appearances observed in different instances are (in the order of intensity) those of inflammation of the uterine peritoneum and ligamenta lata, of the peritoneum, of parenchymatous organs, of the muscular peritoneum; with the production of serum and lymph in large quantities, of blood, of pus, and of coagulable lymph. In the intestinal canal, inflamed patches of the mucous membrane, softening and perforation, and ulceration, are sometimes met with; and the intestines are usually distended with air, or filled with a brownish green fluid, which accumulates from the upper part of the œsophagus to the rectum, and is found to be a modification of bile. The peritoneal coat of the liver is not unfrequently found to be covered with a layer of lymph, when every other part of the peritoneum, except the envelope of the uterus, is sound: its substance is frequently gorged, distended, and softened. When there is much hepatic or uterine lesion, the spleen is found quite broken up, and of the consistence and colour of treacle. Inflammation of the peritoneal coat and substance of the kidneys, with all its consequences, also occurs. The peritoneal coat of the uterus is frequently injected, covered with lymph, or raised by a subjacent layer of pus or blood, or both. Its substance is soft and flabby, and its contractile power destroyed; so that it is as large at ten days after delivery as immediately after the expulsion of the placenta. Small abscesses are found at various depths in its walls; and patches of thoroughly softened and dissolved uterine matter, the softening always commencing on the inner surface. There is often a thick layer of gelatinous blood on the inner surface of the uterus, under which are patches of reticulated lymph, of a greenish brown or modena colour. Dr. Ferguson is satisfied that this is a false membrane, as maintained by Cruveilhier, Dugés, and Seiler, and not the remains of the decidua. The ovaria and fallopian tubes are softened, and deeply injected with blood, serum, lymph, or pus.

In the chest are found, in different examples, copious deposits of turbid serosity and lymph, and all the marks of every stage of disorganization of the lungs. The heart is often enlarged, softened, and friable; and its inner membrane, as well as the great arteries, deeply stained; occasionally lymph and serum are found in the pericardium, and white patches on the outer covering of the heart.

Of the veins, the uterine veins are chiefly affected; their lining membrane often pale, but covered with false membrane or pus. Their coats are thickened, and their cavities more or less obliterated: when the neighbouring veins are affected, the adjacent cellular membrane is hardened or infiltrated, or forms a bed for purulent matter. But the uterine veins are often perfectly healthy, when the spermatic or renal or more distant veins are disorganized. Not only pus and lymph are found in the veins, but gritty and grey or light coagula. The mass of blood frequently retains its fluidity. In some cases some unnatural fluid is found in the vein, without lesion of the vein: whether this is the product of absorption or of inflammation, Dr. Ferguson maintains that puerperal fever is the result of its entering the circulation; and he entertains the opinion that secondary abscesses are the result of new inflammations. Pus may generally be detected, Dr. F. says, in the ligamenta lata, but more frequently in the lymphatics than in the veins; it is collected in small pouches, and gives them a beaded appearance. The constitutional results he regards as being the same.

Although delirium and cerebral disturbance may exist, lesions of the brain are rare in puerperal fever. The limbs are subject to purulent deposits or effusions of serum or blood. According to Dugés, the order of frequency of such attacks, when in the joints or muscles, is, 1, hip; 2, elbow; 3, knee; 4, foot; 5, metacarpus; 6, shoulder. Dr. Ferguson, however, has found the elbow and knee more frequently affected than the hip. When pus is deposited in the muscles, it is rather infiltrated than contained in a cyst; and the muscular fibre is soft and sometimes pultaceous in circumscribed spots. If, instead of pus, serum or sero-sanguinolent fluid is infiltrated into the limb, the appearance is exactly that of erysipelas. The body is rapidly decomposed after death; and Martens affirms that, contrary to the usual course, the internal organs putrefy before the external.

Dr. Ferguson quotes the results as to the relative frequency of the above lesions from Tonellé, founded on 222 dissections, and from Dugés, founded on 341. The general conclusions of M. Tonellé are, that the uterus is more frequently attacked than the peritoneum, by a slight excess in the relative numbers; that these two lesions are mostly combined; that each may in turn fail; and that, in 222 cases, pus was found in the vessels 134 times. In M. Dugés' 341 cases, peritonitis occurred 266 times; and in these the womb was affected in 3 cases in 4. Dr. Robert Lee's report of 45 cases is also referred to; in 32 of which the peritoneum and its appendages were inflamed; in 24 there was uterine phlebitis; in 10 softening of the muscular coat of the uterus; and in 4 pus in the absorbents. We shall here quote a few paragraphs from Dr. Ferguson himself, of which the object is to excite the reader to reflection, and to point out the relative importance of symptoms. His work, indeed, derives no small value from its constant practical bearing; and no better example could be given of the possibility of combining this object with profound and philosophical views of a disease. The day has gone by in which theory was looked upon as no more than idle speculation, unconnected with practice. There are, no doubt, scattered over the country many men, yet surviving in this age of the diffusion of knowledge, who plume themselves on being exclusively practical men; but even these have always some theory of their own, very hastily formed, and never carefully examined. They go on prosperously enough in ordinary cases; but, if their career is watched, not a year passes in which their practical skill is not baffled by some unexpected combination of symptoms, requiring reflection and a knowledge beyond their own experience; and the result is that the patients die; and the friends and the practitioner, the bereaved and the author of the bereavement, unite in the comfortable assurance that nothing more could be done, and in the pious belief that "physicians were in vain."

"If the reader," Dr. Ferguson observes, "have considered the ample and minute table before him, I will at this stage ask in what consists the essence of puerperal fever? and request he will expend some thought on the question before he turns to the chapter in which it is attempted to be discussed.

"I will now merely add a few remarks as to the value of symptoms. It will be seen from my tables that peritoneal pain must be abandoned as a pathognomic and characteristic sign of puerperal fever; since in nineteen cases no such pain was there. In the thirty-three cases detailed at length by Dr. Robert Lee, eight exhibited no peritoneal pain at all. Of the nineteen I have noted, eleven died. Hence the most mortal cases are precisely those where peritoneal pain is absent. By comparing the duration of the attack of pain with the duration of the whole disease, we shall see that peritoneal affection is often of very little value as a mark and sign of danger, or even of the probable length and course of the malady. Where the peritoneal pain is great and sustained, it marks a less dangerous attack; but on this head nothing certain can be inferred, since we cannot measure the action of the complications accompanying it.

"The number of patients who had no pain was 19.

| | | | |
|---|---|---|---|
| Ditto | who had pain for | 1 day, | 51. |
| Ditto | ditto | 2 days, | 48. |
| Ditto | ditto | 3 days, | 22. |
| Ditto | ditto | 4 days, | 18. |
| Ditto | ditto | 5 days, | 6. |
| Ditto | ditto | 7 days, | 5. |
| Ditto | ditto | 8 days, | 4. |

The others had either successive attacks and remissions, or constant tenderness of the abdomen when pressed, showing the existence of morbid action." (p. 48.)

The same tables, he further observes, show that any cerebral disturbance diminishes the chances of recovery; and that the presence of delirium is almost always followed by a fatal result. The presence of intestinal disorder is far less unfavorable, and in epidemics in which it is the most urgent symptom the mortality is very slight. Of 49 cases (out of 204) in which the chest was affected, 30 died; and of 44 in whom there was some deposit in the external parts, 21 died. In 46 cases out of 80 in which the state of the lochia was accurately noted, the discharge was either offensive, scanty, or suppressed in 46. Out of 100 cases observed by Dr. Lee, the lochia were suppressed in 44, and offensive or scanty in 7: these results concurring with those of Dr. Ferguson to show that one half of the cases are complicated with obvious lochial disorder.

The chapter on the Nature of Puerperal Fever is introduced by the consideration of the origin in one source, or in many specific causes, of the manifold affections which have been described, and of the circumstances which regulate their march; for, it is observed, an ophthalmia, an ulcerated joint, an acute peritonitis, may coexist with gangrene in various parts of the body, and be accompanied with fevers of varying character; or these evils may follow in rapid succession. Dr. Ferguson then expresses his own views of the source and nature of puerperal fever in the following propositions:

"1. The phenomena of puerperal fever originate in a vitiation of the fluids.

"2. The causes which are capable of vitiating the fluids are particularly rife after childbirth.

"3. The various forms of puerperal fever depend on this one cause, and may readily be deduced from it." (p. 53.)

In support of the first of these propositions, Dr. Ferguson adduces several experiments performed by Gaspard and Cruveilhier, in which pus, recent and putrid, was introduced into the circulation, in dogs, with results analogous to those which are the product of puerperal fever, in the form called ataxic by Tonellé, and which constitutes Dr. Ferguson's nervous form of the disorder; the peculiar symptoms being produced in such cases, according to the opinion both of M. Tonellé and of Dr. Robert Lee, by the circulation of pus in the veins. From these experiments it is concluded that the vitiation of the fluids will produce fever, with local irritation or inflammation of different organs at the same time. Other experiments, in which the vitiating fluids were introduced in the neighbourhood of the abdomen, and allowed to be absorbed, or directly injected into the veins, are also quoted from Gaspard and from Cruveilhier, to show that peritoneal pain and effusion, suppuration of the eyes, abscesses and disorganization of the muscles, may also be produced by vitiating the blood; and also experiments proving that direct injury to the coats of the veins, causing inflammation and a secretion of fluid or fluids, which become mixed with the circulation, acts in the same manner. (p. 65.) Cases are then alluded to, related by Gordon, Campbell, Kirkland, Baudeloque, and Delamotte, in which retained and putrid placentæ, or coagula, produced a genuine puerperal fever, not distinguishable from the disease above described. Further confirmation of his views may be derived, Dr. Ferguson remarks, from a consideration of the effects of wounds from dissection, the viper-bite, and the effects of some acknowledged diseases of the fluids. The variations in these several instances depend more, he maintains, on the point at which the circulation has begun to be injured than on any other cause. "In puerperal fever the injury is, almost invariably, in the uterus or its appendages: hence the frequent circumscribed peritonitis, metro-peritonitis, and ovarian disorganization." (p. 72.)

Dr. Ferguson proceeds to show, in support of his second proposition, that both mechanical injury of the vessels and their contact with noxious matter, the effects of both of which causes have been already shown to be productive of fever and local consequences resembling those occurring in puerperal fever, are conditions which are combined in the uterus after childbirth. The state of the uterus then, he observes, represents an exact analogy to the surface of an amputated stump, the secondary evils of which he had previously mentioned as so closely resembling those of the puerperal condition; an analogy strongly dwelt upon by Cruveilhier, who however, Dr. Ferguson admits, has somewhat overcharged it; not apparently taking into account the previous preparation for the separation of the fœtus from the mother. In several dissections of the rabbit, Dr. F. has always found the portion of the uterus from which the placenta had been detached perfectly denuded, and the muscular fibres distinctly exposed; the rest of the mucous lining being entire. In the human uterus he found the placental spot equally denuded, but the rest of the mucous membrane hypertrophied, and presenting the appearance of a fine network; which, he conceives, if not shed or cast off, must undergo great changes before it reassumes the appearance of the mucous lining of the uterus in its unimpregnated state. In two instances, where the patients died of puerperal fever, there were only slight vestiges of the membrane, and the inner surface of the uterus was denuded and covered with pendulous flocculi.

Dr. Ferguson's third proposition is but a necessary sequence of the first and second; and it must be conceded to him, whether the cause he assigns be assented to or not, that there is no other assigned cause of which the presence is presumably so constant, whilst some, as peritoneal inflammation and uterine phlebitis, are in many cases absent. There is no difficulty in conceiving the cause assigned by him, a vitiation of the fluids, to act with various intensity, and on various organs, in different cases. The following exposition is highly interesting:

"There are two great systems of venous capillaries: the one of the lung, the ultimate receptacle of all the blood of the body; and the other, in the liver, through which the great mass of blood, which is gathered on the mucous membranes of the intestines, is transmitted. In both of these the blood is subjected to the most important and vital changes; and to effect this, it is largely accumulated in these spots. If the blood were vitiated we should expect, *a priori*, that the liver and intestinal canal, and the lung, would exhibit the most frequent points of disease; and such we have found to be the case in puerperal fever, and in the experiments of Cruveilhier and Gaspard. But how, it will be asked, are we to account for this partitioning off of so diffuse a malady, as that induced by vitiation of the blood? why is it not always spread where-ever there is blood? and why are puerperal fevers, now peritonitic alone, now metro-peritonitic, now gastro-enteric, and now falling on the nervous centres? We know that the blood-vessels, like every other part of the body, are in their nature capable, of themselves, of repairing injury, and stemming the actions of disease. The experiments of Gaspard and Cruveilhier, permit us to infer that there is always an endeavour to hem in the noxious cause as near to the spot first injured as possible; and Mr. Arnott has remarked, what Cruveilhier had stated in 1820, that coagula form, to prevent the spread of inflammation of a venous trunk; while the former gentleman, more minute in his investigation, has always found that the injured vessel is obliterated only up to the first branch it gives off: as if nature, while endeavouring to pen up the exciting cause of malady in as small a compass as possible, wished to use whatever she could of the other channels of circulation. It is from this law, that we find the injuries of puerperal fever so often confined to the uterus and its appendages, to the lower portion of the peritoneum, and to the adjoining intestinal canal, for we have seen that the point of departure for the noxious matter, is in this disease from the veins of the uterus." (p. 82.)

To trace the paths by which distant organs are affected may, as Dr. Ferguson observes, be difficult, because it cannot be ascertained what uterine veins first become affected, or along which branches the infecting matter passes into the circulation, or what is the quantity or the quality of the fluid absorbed; in which respect our knowledge is not greater than that which we possess of other poisons that affect the circulation.

From the action of the poison being more or less confined to the peritoneum, Dr. F. deduces his first, or peritoneal form of the disease; the second or gastro-enteritic, he ascribes to its action on the liver; the third to its impression being principally received by the nervous centres, which impression is not necessarily inflammatory, but rather that condition metaphorically spoken of by John Hunter as "alarm," and which sometimes leads to inflammation. The fourth or complicated form comprehends those cases in which the action of the poison is not confined to certain structures, but diffused by the circulation over many organs.

We must content ourselves with a brief allusion to the admirable chapter on the opinions of authors on the nature of puerperal fever. Dr. Ferguson gives honour due to Dr. Gooch for distinguishing, amidst the conflicting opinions of the time, that there were at least two broadly distinguished forms of the disease; one inflammatory, the other typhoid; but he justly observes, that the distinction is not absolute in every case; the two characters being often variously combined, and the combination demanding a mixed treatment. To Dr. Robert Lee's division,—resting on the assumption that the sole modifier of disease is the organ affected; and according to which, where the peritoneum is inflamed in the puerperal state, the fever is of an inflammatory character; and where the uterine muscular tissue is inflamed, the fever is congestive; and where there is venous inflammation, the fever is typhoid,—it is objected, that there are at least four causes which modify any malady: the producing cause; its quantity or virulence; the organ on which it acts; and the state of the constitution. With reference to M. Tonellé's theory, we find Dr. Ferguson, as we should expect from his enlarged views of disease, repudiating the notion of the puerperal fever being dependent on local inflammation, and rejecting the same phlogistic doctrine as regards ordinary fevers. There are but two systems, he observes, capable of conveying the morbific influence over the frame, the nervous and the vascular; and a rational theory of fevers can only be expected from an investigation of the conjoint action of the two. This view has long been entertained, we imagine, by the majority of pathologists in this country; although defective powers of generalization, combined with an inordinate devotion to morbid anatomy, have so long given currency to more limited theories in the continental schools. Dr. Ferguson has stated the case on both sides with great precision; and does full justice to the unquestionable merits of Broussais, whilst he points out the too great exclusiveness of his doctrine. To this, as indeed to all other parts of his work, we would recommend the careful attention of the student to be directed; not more on account of the general correctness of the author's opinions on the disease which is its object than for the comprehensive and exact principles which are so clearly and eloquently laid down. It is by this combination of the minute study of separate diseases, and a reference of them to the general laws of pathology, that the separate parts of medical knowledge are best improved, and the whole fabric of medicine strengthened. The study of one disease, under a guide like Dr. Ferguson, is an introduction to an acquaintance with many; and to this reading the application of Lord Bacon's observation is truly apt, that it "maketh a full man."

The theory of Ritgen and the greater number of the German writers on this disease, that it is the result of the disturbance of those processes by which the organs, tissues, and fluids, altered by pregnancy, are brought back to their usual state, appears to be imperfect, because if for no other reason, it only comprehends a predisposing cause. At least these recuperative processes must be common in every case, although the disturbance of them may not. Intestinal irritation, with which Dr. M. Hall connects puerperal fever as effect and cause, is not present, Dr. F. remarks, in many genuine cases; and when present is secondary to the "metro-peritonitic" attack; whilst fever, with intestinal irritation, does not produce the lesions found in puerperal fevers. We do not fully see the force of the last clause of these objections, as, of course, Dr. M. Hall's explanation referred exclusively to the puerperal period. Dr. Ferguson admits, that intestinal irritation occasionally becomes, like cold, protracted labour, or mechanical uterine injury, an exciting cause; adding, "so invariably, for example, does a dose of the house medicine (or senna and salts), after causing violent pain and purging, bring on metro-peritonitis, that I have long forborne the use of this drastic irritant, as the routine dose, given on the third day after parturition." (p. 101.) In other circumstances, a dose of senna and salts would scarcely deserve to be stigmatized as a drastic irritant. There must be a peculiar sensibility in the intestinal canal at the time, connected with, though by no means causing, the general disease: but this is only what we should expect.

Among the unexplained circumstances connected with epidemic disorders, it is to be mentioned that there are years in which no puerperal fever appears; and others, as shown by Dr. Ferguson, in which, when fatal here, it is prevalent and fatal in most parts of Europe. Some tables are given which show that the coldest and dampest months are those in which puerperal fever is the most fatal. In twelve years, included in the tabular view, not one death from puerperal fever occurs in the month of July.

The prejudicial effects of the air of hospitals has already been spoken of. A lying-in hospital, Dr. Ferguson says, "should consist either of a series of cottages, or its spacious wards should contain very few patients." If we wished to enforce a greater attention to the subject of public *hygiène* and medical police, we could not do it better than by pointing to the ingeniously objectionable site of the General Lying-in Hospital, as described by Dr. Ferguson; "rather below the level of the river, and surrounded by a mesh-work of open sewers, fifteen hundred feet in extent, receiving the filth of Lambeth."

After noticing the influence of some of the above circumstances on the production of puerperal fever, Dr. Ferguson leads the reader back to the subject of uterine injury, reminding us that he has already stated that there are two sources of the morbid secretion which enters the blood and vitiates it; one the secretion from the uterine wound; the other, injury to any one or more of the uterine sinuses. It is observed, in corroboration, that a considerable number of cases of puerperal fever occur after first labours; that one half of the fatal cases are of this kind; and that the cases of puerperal fever, after instrumental labours, are numerous. The influence of dead children, long contained in utero, is also cited. Hemorrhage and abortions are mentioned as exciting causes; and it is observed that large hemorrhages favour absorption. Unmarried women, and those otherwise depressed in mind or body, as by insufficient food, &c., are predisposed to the disease. The suppression of the lochia, in a great number of the cases, is again spoken of; but the circumstance of the non-occurrence of puerperal fever in many cases, in which the lochia are very offensive, is not, we think, alluded to: it would seem to constitute something like an objection to Dr. Ferguson's theory, unless the degree and quantity are supposed to be the deciding circumstances as to the febrile and metro-peritonitic supervention. We should also have been glad to see more notice taken of the actual or supposed conditions, of a local or general kind, which promote or permit, in the case of the puerperal woman, the occurence of so formidable a disease; while no such results follow the existence, in other cases, of topical affections of an analogous kind, or at least presenting, apparently, equally copious sources of humoral vitiation.

Dr. Ferguson concludes this enquiry into the opinions of others, by acknowledging the services of several previous investigators; Dr. Robert Lee, Velpeau, Dance, Cruveilhier, and before him, Legallois, and previous to them all, Boyer. It is but right to allow Dr. F. to speak for himself:

"I have availed myself," he says, "of the valuable hints and developments contained in these various sources of information, and have endeavoured to prove the views, to compare the facts with each other and with my own, and having arrived at general principles, to see whether these were in their turn capable of serving as an interpretation of the facts of puerperal fever. What I have done, I do not consider as new, but look on as an attempt to demonstrate what has been hitherto a matter of pure conjecture and mere opinion; and so to arrive at that great desideratum, a just theory of this most fatal and most complex malady. I have, in conclusion, to protest against this my attempt being considered as a revival of the follies and errors of humoral pathologists with their four fluid constituents of blood, phlegm, bile, and atrabile; and their cosmic elements, fire, water, earth, and air, their occult causes, and their facile explanations.' It has taken nearly three thousand years to convince physiologists that the whole of a living body is alive, and consequently subject to all the impressions and reactions of the vital power. At first the fluids were the sole seat of life, and then the solids became exclusively gifted; and each hypothesis furnished the root of a branching nosological tree. Latterly, the best modern observers have traced much of disease and morbid formation to disorder in the fluids. The danger is not from a rational humoral pathology, but from a retrogression to the old irrational one: not from the cautious limitations of Andral and Cruveilhier, but from the very able but somewhat exclusive doctrines of Stevens. Another objection may be further urged against my views as to all the varieties of local lesion being consecutive of a vitiated circulation. As peritonitis, enteritis, or metritis, may occur in the unimpregnated woman primarily, why are we to believe that in the impregnated alone they are mere secondary derivatives? To this I would answer, that I by no means deny that any tissue or organ may be primarily attacked in both states of the female system. That there may be a peritonitis, which is not the result of a puerperal fever, as there may be a pneumonia or congested brain, not the effect of a typhus. But I am quite certain, these purely local inflammations with symptomatic fevers, are very rare after childbirth, and that no one can fail to see a vast difference in the formidable accompaniments of the local inflammation after childbirth, and those which occur before it." (p. 110.)

A large portion of Dr. Ferguson's volume is devoted to the *treatment* of puerperal fever; and this is copiously illustrated by cases which we shall not attempt to condense. Those who wish to become prepared for the serious responsibilities of puerperal practice will do well to peruse this portion of the book with scrupulous attention. Whoever reflects upon the nature of those responsibilities, the difficulty of diagnosis, the obscurity of indication, and the importance of a prompt and correct decision, will not easily think that enough has been done, even with very careful study, to be secured from mistake, and protected from the painful conviction of having fallen into fatal error. In many diseases, error may be retrieved, and life saved; but in this formidable malady, a wrong opinion, an improper system of practice, will be too speedily followed by bad consequences to be repaired. If death holds his dart aloft, shakes it, and delays to strike, it is merely to afford the practitioner one chance of success; and if that is missed, no future care, no anxious intercession, can avert his weapon more. In almost every epidemic of this nature, it appears that one in every three who are attacked dies; a mortality equal to that of the malignant cholera: and, as Dr. Ferguson expresses it, "to save two out of three may be termed good practice in an epidemic season." (p. 112.) Indeed, although Dr. Armstrong had to contend with a disease in which he was enabled to *cure* forty out of forty-four; Dr. W. Hunter *lost* thirty out of thirty-one cases in another epidemic; and in the Paris hospitals, in 1746, *not one* recovered.

*Treatment of the peritoneal form.* The first application which Dr. Ferguson mentions is that of a large linseed-meal poultice, applied over the whole abdomen, made sufficiently thick to retain warmth for four hours: it soothes the pain; it induces copious perspiration; it induces sleep. If general bleeding does not seem required, and, on the other hand, the symptoms are not typhoid, ten grains of Dover's powder may be given as soon as the poultice is applied. Probably as much advantage would be found to ensue from a bran-poultice (bran in a flannel bag dipped in hot water), and the application is more convenient and cleanly. In four hours, if the symptoms are alleviated, a fresh poultice may be applied, and the Dover's powder repeated: but if within four hours of this second dose, the symptoms are not improving, depletion must be at once resorted to. The efficacy of this plan depends on its early application: and, in cases in which the necessity of venesection is not obvious, the delay of it for six hours, Dr. Ferguson says, is not injurious, nor the use of the opiate hurtful. On this point, although not without trembling, we must defer to Dr. Ferguson's experience. In such an assurance, from one so experienced and thoughtful, there is certainly no little consolation. The inconvenience of diminishing the secretions by the opiate may be obviated by combining it with a mercurial aperient. One fifth of Dr. Ferguson's cases were treated in this way, without bleeding or leeching, and of these only two died. This section is illustrated by twenty-two cases, which, with the remarks interspersed, we necessarily, but with reluctance, pass over. We trust, however, that there is not a study or a surgery in which they will not be read. They are arranged with the intention of showing that the intensity of the malady is chiefly expended on the peritoneum; that there is every variety of degree of this intensity; and that some are best relieved by acting "on the nervous element of inflammation," while others also require depletion, to every variety of extent. On this vital point of practice, we must again quote Dr. Ferguson's own words:

"The following is the sum of my own experience of bleeding as a remedy in puerperal fever: of all the means we possess of arresting this malady, I believe bleeding, general or topical, to be by far the most extensively applicable. The cases in which it is not so are exceptions to the rule. Mercury, turpentine, emetics, opiates, sudorifics, &c., have a more limited range of utility than abstraction of blood. But while I admit this, I am equally certain, that *large* bleeding has not been borne in this malady, generally speaking, during the last twelve years. Those who have borne it best and required it most were—1. Those who were originally vigorous, and in whom no chronic ailment of the intestinal canal or lungs previously existed. 2. Those in whom the fever was accompanied by a general turgor of the frame: their aspect being that of a person who has been flushed by running, and forming a marked contrast with the pinched, shrivelled, and stricken looks of those labouring under the typhoid form of the malady. 3. Those in whom the disease seemed to be limited to one organ. It may be asserted, with more hesitation, however, that they who are confined out of an hospital, exhibit greater reactive powers than those who are confined in one. The pulse, as Gordon has remarked, is very deceptive; and the cases I have given show that painfulness is no sufficient criterion of the necessity of depletion.

Besides these general indications, puerperal fever has invariably the character common to the ordinary fevers raging with it: if the latter require depletions, the presumption is, that the former will also. It is curious that, in the majority of cases bled, the blood is neither cupped nor buffed. The persons who do not bear large bleedings are those attacked by the ataxic or gastro-enteric forms; even though they be of originally strong constitutions: also those labouring under the complicated form, where many organs are, simultaneously, under the grasp of the diffusive malady. But in this last class there are so many shades of disease, that no absolute rule can be laid down. If large bleeding be determined on, it must, to be beneficial, be resorted to within the first twenty-four hours of the attack. In the second stage of the disease it is often rapidly fatal. If the bleeding be made early, it may be often repeated. It appears, where it does not remove the malady, to stop its progress, and make it continue lingering in its first stage, so that the repetition of venesection is late, only as to the lapse of time, but not tardy, as to the progress of the disease. Of local depletion by leeching, it may be said that the cases, in all the four forms of puerperal fever, are very few indeed, which do not permit us to resort to it. It often removes a pain which will not yield to bloodletting. The number of leeches required will, of course, vary with the case; in some, six is sufficient, in others, six dozen will scarcely be so. But the average cases will require from two to three dozen." (p. 152.)

The application of leeches to the uterus itself, by means of a tube, or by simply introducing them into the vagina, is strongly recommended "in the less overwhelming forms" of the malady; from six to twenty may be safely and beneficially used.

*Treatment of the gastro-enteric form.* Active purgatives, which have been recommended by some authors in this form of the disease, are considered by Dr. Ferguson as decidedly mischievous, and our own experience leaves no doubt of this in our mind. If the bowels require to be freed from scybala, the aperient used should be, not the senna and salts of which the bad results have been already mentioned, but castor oil, with the addition of the tincture of hyoscyamus or hop, in a full dose, to prevent griping. General bleeding is seldom required in this form; topical bleedings may be serviceable; but even these often produce fainting. It must not be forgotten, Dr. F. says, that in this form of the disease the nervous system is much wrought on, and that there is another cause of debility in the intestinal flux. The treatment advised is the moderate application of leeches, so as to reduce the disease to a simple fever with gastro-enteric irritation. If the skin is early dusky, and there is nausea or vomiting, an emetic is to be first given. If there is no nausea or vomiting, but intestinal flux, with a red tongue smeared with saburra, a large dose of calomel (from ten to fifteen grains) is recommended; of which the effect will be several large pultaceous stools; and the tongue will become clean, less red, and moister, and the pulse less frequent. Sometimes a repetition of this dose is only required for the cure; but in other cases, diarrhœa recommences and soon becomes colliquative; in which state Dover's powder, with a mercurial, is serviceable if the secretions are diseased as well as copious; or, if not, absorbents and astringents are required. If there is great debility, wine must be freely given, in gruel or sago; but if it is not so great, the strength may be supported by soups, *thickened* with any gelatinous substance. If delirium, or nightmare, or fantastic visions torment the patient at night, a full dose of Battley's sedative, in camphor mixture, with a few additional grains of camphor, may be given. When the diarrhœa abates, tonics may be given, and "these are always better borne when ammonia is a chief ingredient." (p. 160.)

*Treatment of the ataxic forms.* From amidst the profound observations by which this section, comprehending cases connected with the most hidden operations of the nervous system, is introduced to the reader, we cannot resist transcribing, in reference to the peculiar state of weakness of that system with which the ataxic form of puerperal fever is associated, the following most happy and eloquent reference to one of the greatest physiologists who ever adorned this or any other country.

"This state of the nervous system was, as far as I know, first noticed and made a prominent principle for guidance in practice, by John Hunter, and received from him the remarkable designation of 'action without power;' one of those apparent paradoxes, which never could have arisen but in the mind of one who had been long accustomed to commune with the deeper intellect displayed in nature, and who, seeking in vain among the symbols of human thought for an adequate expression, was forced to create a language for ideas to which he alone could attain. There is such a remarkable striving throughout the whole of his essay on the blood, to make this thought intelligible and available, that it is impossible not to feel the importance he attaches to it. Several cases are scattered through the work, intended to unveil the mystery, and to show how largely this peculiar form of debility, which imitates all the acts of genuine inflammation, and yet has none of its power, enters into the domain of disease. The expressions which Bichat, and others, have coined, as excitability, irritation, are but feeble and marrowless, and require the volumes they have written to illustrate their obscurity; while the pithy sentence of Hunter is at once a discovery of the essence of the malady, and a guide to the practice." (p. 177.)

The practice in this form of the disease is, the reader will readily conclude, "to sustain." Stimuli, largely and frequently given, are necessary, where there is sinking; and, where there is no visible sinking, sedatives, but especially Battley's laudanum, must be immediately given. Nourishing food and tonics become subsequently appropriate. Dr. Ferguson introduces the striking case from Gooch, in which that eminently sagacious practitioner was sent for to see the wife of a physician, who had suddenly become apparently affected with phrenitis; a hot and turgid head, a quick pulse, and raving. The husband proposed a large bleeding and rapid salivation. Dr. Gooch begged permission to try the efficacy of "a dose of Battley"; forty drops procured tranquil sleep, from which the patient awoke debilitated, but quite rational and continued so.

There are cases, even of the ataxic form, in which, the head being much flushed and there being pain which the sedative has not removed, it is necessary to apply leeches; but even this must be done watchfully and cautiously. This section of the treatment, and the preceding one, are also, like the first, illustrated by cases briefly related, and which will well repay the patient reader.

*Treatment of the complicated form.* Of this, the slighter cases are alone curable. Fortunately, although it generally accompanies each epidemic, it rarely forms the general epidemic character. Quoting Cruveilhier's discouraging attempt to answer a question which must, in some period or other of every medical man's life, have presented itself to his mind under distressing circumstances,—"what treatment shall we oppose to purulent infection?"—Dr. Ferguson attempts, with great modesty and candour, to ascertain, amidst the difficulties, some landmarks to guide the practitioner in respect to the various means so often indiscriminately and uselessly resorted to; mercury and quinine, for example; emetics and stimuli; turpentine and sudorifics; vesicatories and strong diuretics. He reminds us, that the purulent infection is attended with various states of the constitution in different epidemics, and in different persons; requiring in some, in consequence of great re-action, depletion; whilst others will sink at once: and that between these extremes every variety may be expected, dependent on the organs attacked, and the degree in which they suffer. So that the indications, he observes, are to attend to the local lesions, but not to forget that these are but the effects of a more diffusive cause.

These principles are illustrated by particular remarks on emetics, purgatives, mercurials, turpentine, and Stephens' mixture. In the year 1782, Doulcet, physician to the Hôtel Dieu, obtained the greatest success from emetics (ipecacuanha); he was largely compensated; but in the following year this mode of treatment proved quite unsuccessful. The practical question is, Dr. Ferguson observes, what are the cases in which this remedy is applicable? M. Doulcet was led to his practice from the observation of spontaneous vomiting in a patient at the very commencement of the attack, and this, Dr. F. thinks, furnishes an answer to the question. When the violence of the malady has fallen on the liver, and there is early nausea and spontaneous vomiting, emetics may be useful. For some cases treated in this manner we must refer the reader to his book. The remarks on purgatives, and on the cases requiring them, and their adaptation to the character of the case to be treated, are highly judicious. The section on the employment of mercurials contains some valuable observations on their antiphlegmonous properties, from lectures delivered by Dr. Farre in 1826, which we shall not attempt to abridge. Of turpentine, Dr. Ferguson has no experience in puerperal fever: he believes it might be useful in the second stage of the disease, when the intestines are tympanitic; and he has given forty drops in syrup, or emulsion, made of yolk of egg, every four hours. The only benefit he has observed is that it strengthens the pulse. It is beneficial as a rubefacient, and Dr. F. appears to think that vesicating agents are most useful in this malady when their operation is limited in this point. He has tried, without benefit or injury, the use of the celebrated briny mixture of Dr. Stephens.

The following observations are on points of practice too important to be omitted:

"In the complicated form of puerperal fever, the 'deposits' which take place in the limbs and eye, require much attention in the treatment. If there be constitutional vigour, or if the part be affected early in the malady, leeches may be applied. They are contra-indicated when these disorganizing processes appear in frames enfeebled by disease or constitutional causes. Two or three examples are recorded in my tables, where a few leeches applied, late in the disease, caused immediate sinking. The local inflammation is, even in the very last moments of life, exceedingly painful, and seems to demand depletion; but, unless the whole state of the patient be taken into the account, a dozen of leeches will turn the vibrating scale from life to death. It is when the eye is attacked that leeching will be oftenest useful. When the seat of deposit is in the cellular and muscular portions of the limb, it should be covered either with a linseed-meal poultice, or with flannels soaked in decoction of poppy and chamomile flowers. The ease obtained is very great; the swelling subsides in many instances entirely, leaving the limb unscathed; in others it is removed in every part but two or three spots which are found to be puffed out with pus, which should be evacuated. Where deposition takes place in a joint, the treatment of leeching and poulticing, will sometimes arrest its disorganization: in all it will give ease. But there is a tardy convalescence to be looked for, and, even with the best surgical attention, it is often impossible to prevent the loss of motion of the affected limb." (p. 226.)

But we must here close our notice of this valuable practical work, leaving many observations of much interest without comment, for with such almost every page abounds. In the Appendix are inserted an account of Mr. Gulliver's Researches on Suppuration; a letter from Dr. Copland on the Treatment of Puerperal Fever in Queen Charlotte's Lying-in Hospital; and one from Dr. Watson, professor of medicine in King's College, on the Use of Opium in certain Inflammatory Diseases; besides other articles bearing on important points in relation to the general subject of the work.

Appended to the essay on Puerperal Fever is a lecture by Dr. Ferguson, delivered by him on opening the medical session in King's College in October, 1836. The thanks of the profession are, we think, due to Dr. Latham and Dr. Watson, by whose advice Dr. Ferguson was induced to print this masterly discourse; which, whilst it touches on many points of philosophical import and general interest, bears strongly and throughout on the improvement of medicine.—Our opinion of the merit of Dr. Ferguson's Essay has been several times incidentally expressed when noticing different parts of it. He is one of those writers whose works not only tend to improve the science of medicine and to render it more useful as a practical art, but whose writings add to the stores of its most valuable literature. His style is clear and forcible, unaffected and elegant. The minutest points of practice are spoken of with precision and distinctness; his narrations are perspicuous; and whenever he touches on the higher branches of physiological enquiry, he does so in the manner of one who does not approach such topics rashly, but has often and deeply reflected on the limited time and faculties with which man attempts to pick up a few pebbles of knowledge by the great sea of truth that lies before him. There are to whom these things are of small account. We are not of the number; but rather say, in the words of an ancient master, if not of medicine, of philosophy: "Non quærit æger medicum eloquentem sed sanantem: si tamen ita competit, ut idem ille qui sanare potest, compte de iis quæ facienda sunt, disserat, boni consulet." (*Senec. Epist.* 76.)

---

